# Pleiotropic Roles of Cholesteryl Sulfate during *Entamoeba* Encystation: Involvement in Cell Rounding and Development of Membrane Impermeability

**DOI:** 10.1128/msphere.00299-22

**Published:** 2022-08-09

**Authors:** Fumika Mi-ichi, Hiroshi Tsugawa, Makoto Arita, Hiroki Yoshida

**Affiliations:** a Division of Molecular and Cellular Immunoscience, Department of Biomolecular Sciences, Faculty of Medicine, Saga University, Saga, Japan; b Central Laboratory, Institute of Tropical Medicine (NEKKEN), Nagasaki University, Nagasaki, Japan; c Department of Biotechnology and Life Science, Tokyo University of Agriculture and Technology, Koganei, Tokyo, Japan; d Laboratory for Metabolomics, RIKEN Center for Integrative Medical Sciences, Yokohama, Kanagawa, Japan; e Graduate School of Medical Life Science, Yokohama City University, Yokohama, Japan; f Division of Physiological Chemistry and Metabolism, Graduate School of Pharmaceutical Sciences, Keio University, Tokyo, Japan; University at Buffalo

**Keywords:** amoebiasis, parasitology, dormancy, lipid metabolism, membrane property control, morphogenesis, infectious disease

## Abstract

Entamoeba histolytica, a protozoan parasite, causes amoebiasis, which is a global public health problem. The major route of infection is oral ingestion of cysts, the only form that is able to transmit to a new host. Cysts are produced by cell differentiation from proliferative trophozoites in a process termed “encystation.” During encystation, cell morphology is markedly changed; motile amoeboid cells become rounded, nonmotile cells. Concomitantly, cell components change and significant fluctuations of metabolites occur. Cholesteryl sulfate (CS) is a crucial metabolite for encystation. However, its precise role remains uncertain. To address this issue, we used *in vitro* culture of Entamoeba invadens as the model system for the E. histolytica encystation study and identified serum-free culture conditions with CS supplementation at concentrations similar to intracellular CS concentrations during natural encystation. Using this culture system, we show that CS exerts pleiotropic effects during *Entamoeba* encystation, affecting cell rounding and development of membrane impermeability. CS dose dependently induced and maintained encysting cells as spherical maturing cysts with almost no phagocytosis activity. Consequently, the percentage of mature cysts was increased. CS treatment also caused time- and dose-dependent development of membrane impermeability in encysting cells via induction of *de novo* synthesis of dihydroceramides containing very long *N*-acyl chains (≥26 carbons). These results indicate that CS-mediated morphological and physiological changes are necessary for the formation of mature cysts and the maintenance of the *Entamoeba* life cycle. Our findings also reveal important morphological aspects of the process of dormancy and the control of membrane structure.

**IMPORTANCE** Entamoeba histolytica causes a parasitic infectious disease, amoebiasis. Amoebiasis is a global public health problem with a high occurrence of infection and inadequate clinical options. The parasite alternates its form between a proliferative trophozoite and a dormant cyst that enables the parasite to adapt to new environments. The transition stage in which trophozoites differentiate into cysts is termed “encystation.” Cholesteryl sulfate is essential for encystation; however, its precise role remains to be determined. Here, we show that cholesteryl sulfate is a multifunctional metabolite exerting pleiotropic roles during *Entamoeba* encystation, including the rounding of cells and the development of membrane impermeability. Such morphological and physiological changes are required for *Entamoeba* to produce cysts that are transmissible to a new host, which is essential for maintenance of the *Entamoeba* life cycle. Our findings are therefore relevant not only to *Entamoeba* biology but also to general cell and lipid biology.

## INTRODUCTION

Entamoeba histolytica, a protozoan parasite belonging to the phylum Amoebozoa, causes amoebiasis, for which new therapeutics are urgently needed because of limited clinical options (1, 2). As a parasitic strategy, E. histolytica alternates its form between a proliferative trophozoite and a dormant cyst (3, 4). Cysts differentiate from trophozoites via stage transition, which is termed “encystation” (5). Encystation is a fundamental cell differentiation process, and the change in cell morphology is obvious; motile amoeboid cells become rounded nonmotile cells. Concurrently, substantial changes in cell components occur, and a series of metabolic pathways are coordinately activated or inactivated to produce the metabolites necessary for encystation (3, 6, 7). The cyst is the only form able to transmit to a new host; therefore, *Entamoeba* encystation is an important subject from a medical as well as a biological perspective (3). Until recently, E. histolytica was known not to encyst after adaptation to *in vitro* cultures (8); therefore, *in vitro* culture of Entamoeba invadens, a reptilian parasite, has been used as a model system for encystation studies (3, 9).

We previously showed that cholesteryl sulfate (CS) plays an important role in *Entamoeba* encystation. CS is a terminal product synthesized specifically during encystation via sulfolipid metabolism (10). *Entamoeba* sulfolipid metabolism consists of two processes localized in different cell compartments. One is mitosomal sulfate activation, which produces 3′-phosphoadenosine 5′-phosphosulfate (PAPS) by sequential reactions catalyzed by ATP sulfurylase (AS) and adenosine 5′-phosphosulfate kinase (APSK). The other is cytosolic lipid sulfonation, in which the sulfate moiety of PAPS is transferred to an acceptor lipid by sulfotransferases (11). Addition of CS to *in vitro* cultures of *E*. *invadens* dose dependently elevates the number of cysts formed (10). Conversely, treatment of cultures with chlorate, an AS inhibitor, or auranofin, an APSK inhibitor, dose dependently impairs cyst formation via inhibition of sulfolipid synthesis including CS (10, 12). Hence, these lines of evidence indicate that CS is a crucial metabolite for *Entamoeba* encystation. However, the precise role of CS remains to be revealed. To address this issue, we estimated the intracellular concentrations of CS in *in vitro* cultured *E*. *invadens* trophozoites and mature cysts to be 243 ± 37 μM and 539 ± 37 μM, respectively. We then explored conditions that enable the effect of CS on *E*. *invadens* cells to be investigated, and we identified culture conditions with 100 to 500 μM CS supplementation that mimic part of the natural encystation process (at least up to ~24 h postinduction). Using these conditions, we elucidated the roles of CS in the process of *Entamoeba* cyst formation.

## RESULTS

### CS initiates encystation cell rounding and decreased membrane permeability.

During the early phase of *Entamoeba* encystation, CS is specifically synthesized in the cytoplasm (10, 13). Supplementation of CS into a standard encystation-inducing culture medium (44% LG medium (14) supplemented with 3% adult bovine serum [ABS]), which presumably results in elevating CS levels in encysting cells, enhances the cyst formation efficiency of *E. invadens* (10). To determine changes in CS levels during the *Entamoeba* life cycle, we measured intracellular concentrations of CS in *E*. *invadens* trophozoites and mature cysts by targeted liquid chromatography-tandem mass spectrometry (LC-MS/MS) using *in vitro* cultures. CS concentrations were estimated to be 243 ± 37 μM in trophozoites and 539 ± 37 μM in mature cysts. The packed cell volumes of trophozoites and cysts (1 × 10^6^ cells each) were estimated as 20 and 10 μL, respectively. Considering that the CS concentration range in human plasma is 3.7 to 6.9 μM (15), it is likely that *Entamoeba* itself provides the CS required for encystation using cholesterol from the host because *Entamoeba* lacks a *de novo* cholesterol synthesis pathway (16). To investigate the role of CS in *Entamoeba*, CS at concentrations ranging from 100 to 500 μM, which resembles the rise in CS concentration during *Entamoeba* encsytation, was added to 44% LG medium supplemented with 15.8 mM glucose without serum (modified LG [–]), a cholesterol (the precursor of CS)-free medium.

**FIG 1:**
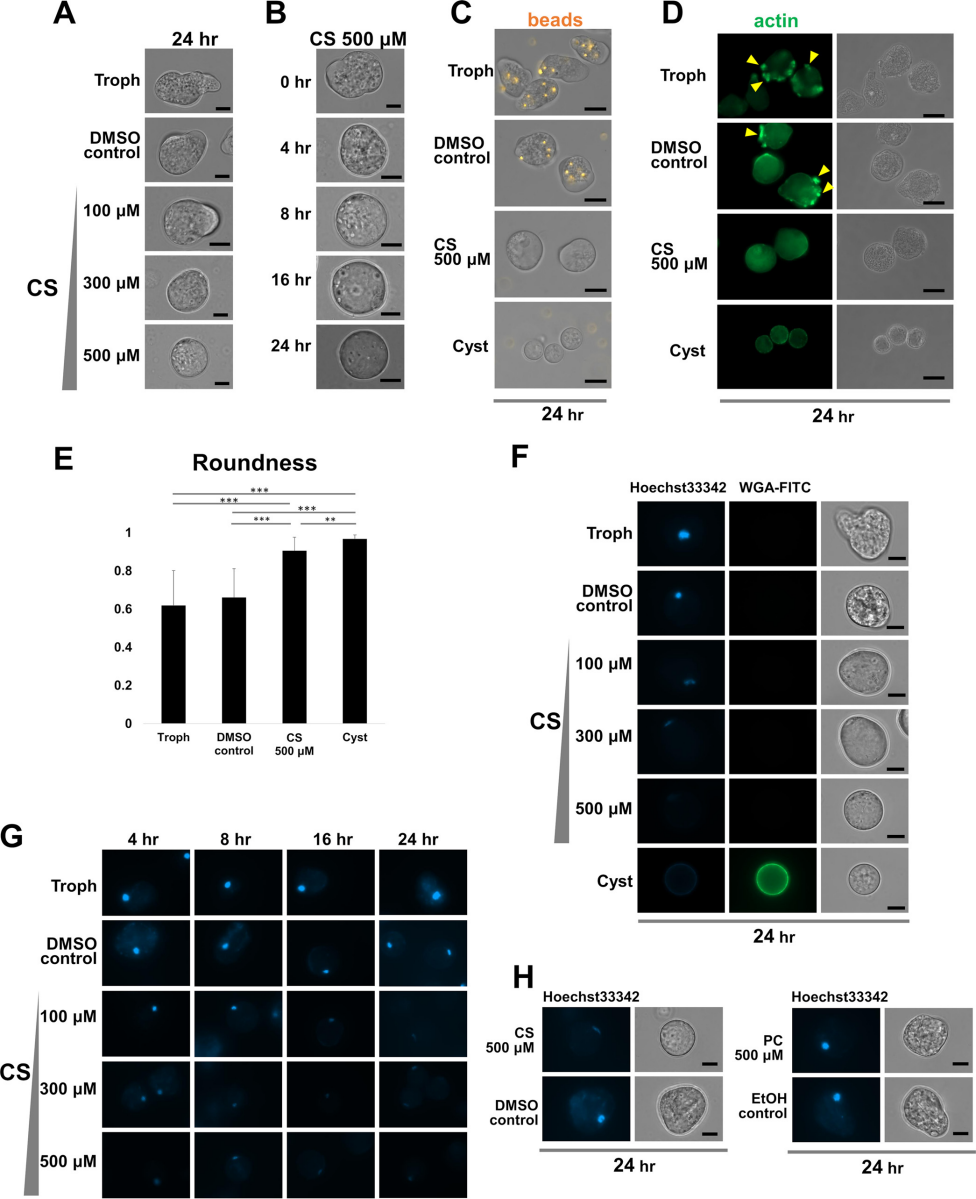
CS initiates cell rounding and decreased phagocytic activity and membrane permeability in encysting *Entamoeba* cells. (A) CS dose dependently caused rounding of encysting cells. (B) CS-mediated cell rounding was time dependent. (C) CS-treated cells lost the ability to ingest latex beads. (D) Actin filaments were visualized with Alexa Fluor 488 phalloidin. Phagocytic cups are indicated by yellow arrowheads. (E) Cell roundness. A total of 20 CellTracker-stained cells randomly selected from each culture were analyzed using ImageJ software (https://imagej.nih.gov/ij/). The value of roundness was calculated for each cell and is shown as averages (bars) with standard deviations (SDs) (error bars). **, *P* < 0.01; ***, *P* < 0.001 (two-tailed unpaired Student’s *t* test). (F) CS dose dependently decreased encysting cell membrane permeability. (G) CS time and dose dependently decreased encysting cell membrane permeability. (H) PC did not cause encysting *Entamoeba* cells to become round or decrease their membrane permeability. Bar indicates 10 μm. Representative data are shown from three independent experiments in panels A, B, F, and G or from two independent experiments in panels C, D, E, and H.

Modified LG (–) maintained *E. invadens* cells as amoeboid, trophozoite-like cells for at least 24 h. Immediately after encystation induction, CS was added to modified LG (–), and 24 h later, encysting *E. invadens* cells became rounded in a dose-dependent manner (Fig. 1A). Treatment with 500 μM CS time dependently made cells become as spherical as mature cysts (Fig. 1B). During the time course of *Entamoeba* cyst formation, phagocytosis decreases in the cells. Consistently, latex beads were not taken up by normal encysting cells but were clearly incorporated into trophozoites (Fig. 1C). Latex beads were also not taken up by 500 μM CS-treated cells, indicating that CS-treated cells act like encysting cells. Furthermore, phagocytic cup formation was only seen in trophozoites (Fig. 1D; 17). Although phagocytic cups were not formed by CS-induced differentiating cells or normal encysting cells, CS-treated cells showed localization of actin distinct from that in normal encysting cells in which polymerized actin was only observed in the cortical region (dense signals in cyst in Fig. 1D), suggesting that certain cytoskeletal changes take place in CS-treated cells and are delayed or advanced compared to those in encysting cells. CS-treated cells also appeared more round than dimethyl sulfoxide (DMSO)-treated control cells and trophozoites but a little less round than cysts (Fig. 1E). Furthermore, similar to mature cysts, CS-treated cells had decreased membrane permeability even though, unlike mature cysts, the cell wall was not synthesized (Fig. 1F). The CS-mediated decrease in membrane permeability, which was monitored by Hoechst 33342 uptake, was dose and time dependent (Fig. 1F and G). In contrast, treatment with 500 μM phosphatidylcholine (PC) did not induce these cellular changes (Fig. 1H). These results together with the previous finding that CS synthesis is stage specifically induced in encysting *Entamoeba* cells (10) indicate that upon reaching an optimum intracellular CS level, cells initiate becoming sphere shaped and decrease phagocytotic activity and membrane permeability.

### CS maintains the spherical shape of encysting cells.

**FIG 2:**
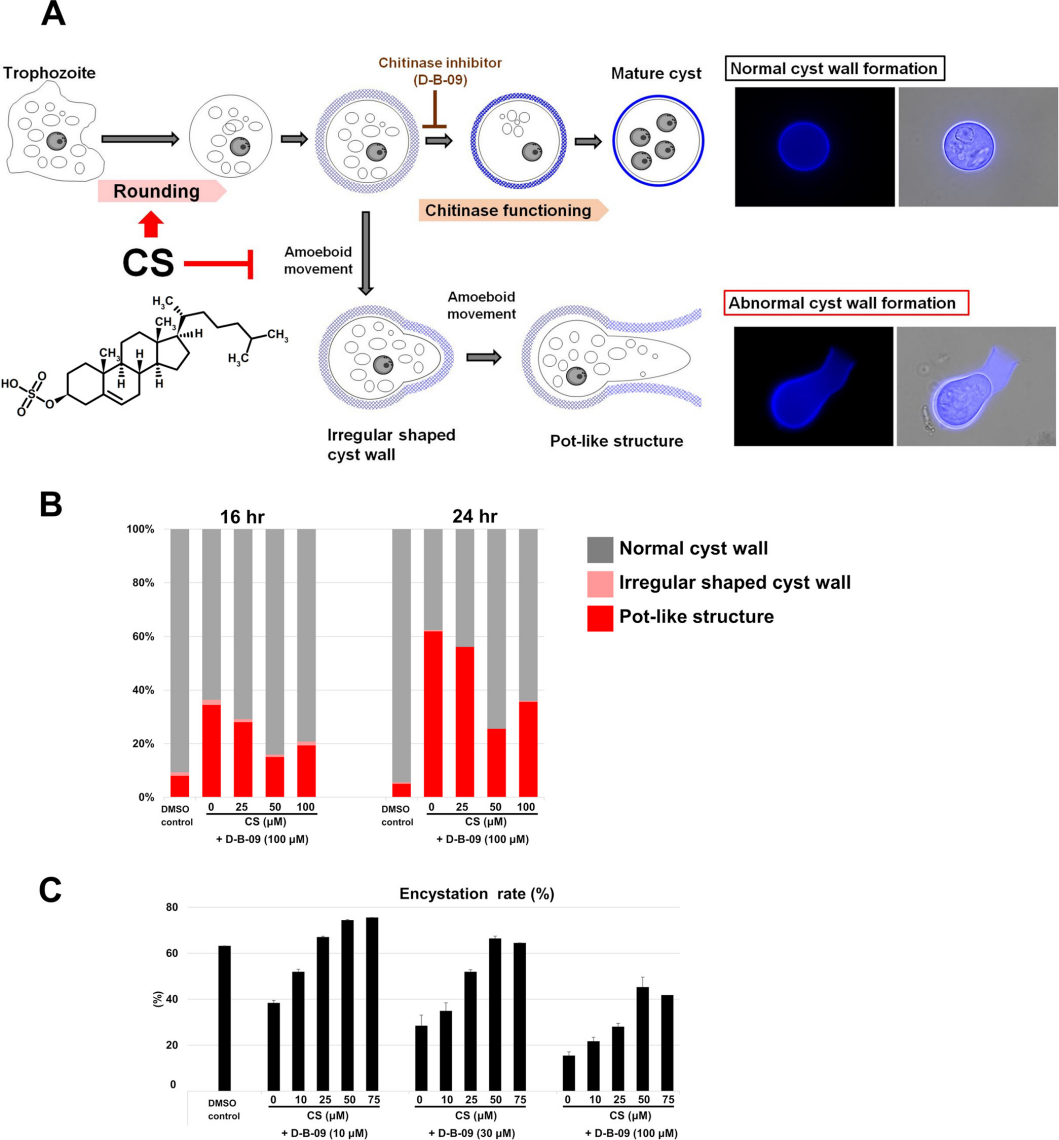
CS prevents the formation of abnormal cyst walls induced via chitinase inhibition, as in pot-like structures, by maintaining encysting *Entamoeba* cells in a spherical shape. (A) Schematic illustration of CS roles during *Entamoeba* encystation. Images show calcofluor-staining (left) and overlay of calcofluor-staining and bright-field images (right). (B) CS dose dependently canceled the enhancement of chitinase inhibitor (D-B-09)-mediated pot-like structure formation. Shown is a stacked bar graph of percentages of normal, irregular shaped, and pot-like cyst walls, detected 16 and 24 h after induction of encystation in a standard encystation-inducing culture in the presence of 100 μM D-B-09 together with various concentrations of CS (0 to 100 μM) or absence of D-B-09 (DMSO control). At each time point, 200 calcofluor-fluorescent cells were randomly selected, and the number of cells with each structure was manually counted. Representative data are shown from three independent experiments. (C) CS dose dependently restored encystation efficiency, which was decreased by D-B-09 treatment. Encystation rates at 72 h postinduction in different culture conditions were determined by flow cytometry. *E. invadens* was cultivated in a standard encystation-inducing medium supplemented with various concentrations of D-B-09 (10, 30, and 100 μM) in the presence of CS (0 to 75 μM) or without D-B-09 (DMSO control). Representative data are shown as means (filled bars) with variation from the mean (error bars) from duplicates of three independent experiments.

Becoming a sphere-shaped cell is one of the most prominent phenomena of *Entamoeba* cyst formation. Recently, we showed that abnormal cyst wall formation, the most characteristic being a pot-like structure, naturally occurs during *Entamoeba* cyst formation and that chitinase inhibition enhances this abnormal cyst wall formation. We also assumed that pot-like structures result from amoeboid movement of encysting cells, which are surrounded by incomplete cyst walls (see Fig. 2A; 18). We therefore hypothesized that maintaining the spherical shape of encysting cells is a prerequisite to the formation of a rigid cyst wall and becoming round mature cysts. To assess this hypothesis, we tested whether addition of CS reduces the generation of pot-like structures. Different concentrations of CS (0 to 100 μM) were added to a standard encystation-inducing culture medium (44% LG medium supplemented with 3% ABS) containing the chitinase inhibitor, D-B-09, at 100 μM, which enhances the frequency of pot-like structure cyst wall formation (18).

Addition of CS dose dependently decreased the percentage of pot-like structures at 16 h postinduction; conversely, the percentage of round mature cysts increased. This trend was also observed at 24 h postinduction (Fig. 2B). Importantly, addition of CS to assay cultures, into which different concentrations of D-B-09 were supplemented, all dose dependently restored the encystation rate at 72 h postinduction (Fig. 2C). As was previously observed (10), beyond the optimum concentration, CS showed a toxic effect on encysting cells (at 100 and 75 μM in Fig. 2B and C, respectively). These results indicate that CS treatment maintains encysting cells in a spherical shape and arrests the generation of pot-like structures, resulting in an increased percentage of mature cysts (Fig. 2A).

### CS functionally interacts with very-long-chain Cer-NDSs to develop membrane impermeability in encysting *Entamoeba* cells.

During *Entamoeba* cyst formation, membrane impermeability is generated to render the cell resistant to environmental assault, such as desiccation (19–21). Recently, we showed that dihydroceramides (Cer-NDSs) containing very long *N*-acyl chains (≥26 carbons) are indispensable for developing membrane impermeability during *Entamoeba* encystation. Three ceramide synthase genes (*CerS2*, *5*, and *6*) are responsible for their synthesis and are specifically upregulated during encystation (22). We therefore assumed that in encysting *Entamoeba* cells, CS functionally interacts with very-long-chain Cer-NDSs to generate membrane impermeability.

**FIG 3:**
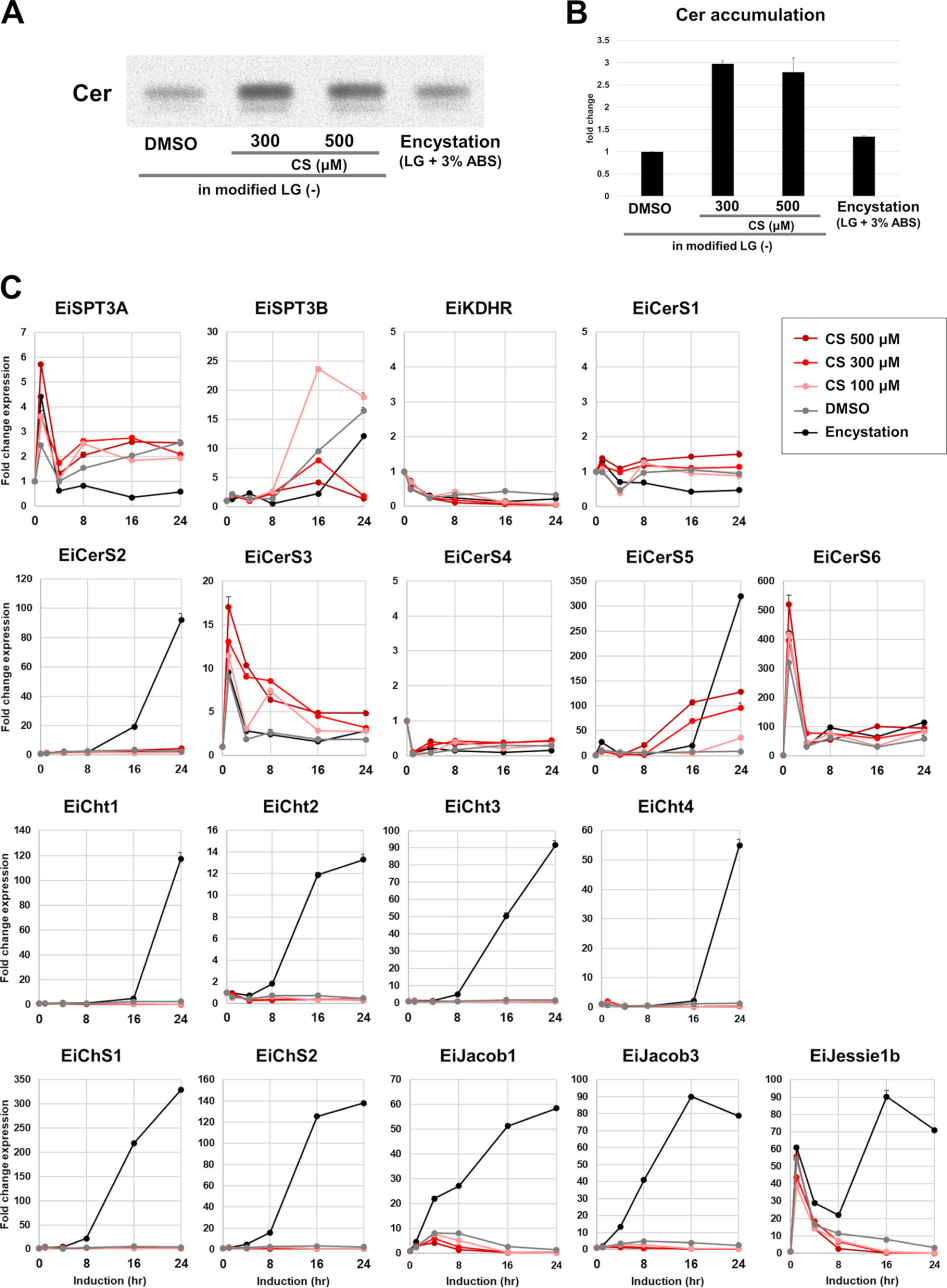

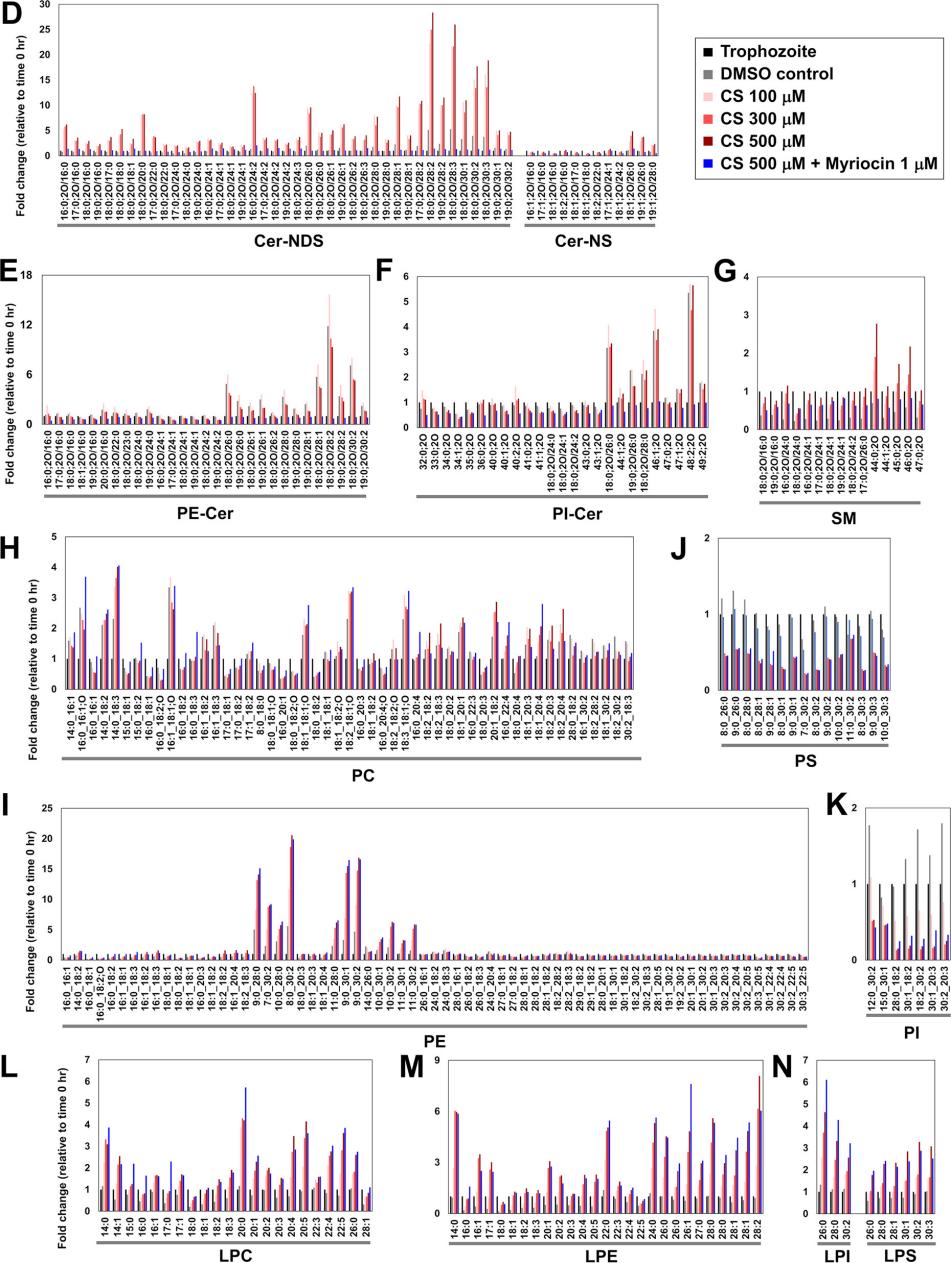
CS elevates *de novo* synthesis of very-long-chain dihydroceramides during *Entamoeba* cell differentiation. (A) Ceramide accumulation in differentiating cells. Thin layer chromatography of lipids extracted from encysting cells or CS-induced differentiating cells that were metabolically labeled with ^14^C-serine. Representative data are shown from two independent experiments. (B) The accumulation of Cer in CS-induced differentiating cells and normal encysting cells for 24 h is shown as the fold change relative to the level in DMSO-treated control cells (*n* = 2). Values were calculated from two independent experiments and are shown as averages (filled bars) with variation from the mean (error bars). (C) Transcriptional changes of the genes encoding the enzymes involved in *de novo* ceramide synthesis (EiSPT3A, B, EiKDHR, and EiCerS1-6) and chitin metabolism (chitinase [EiCht1-4] and chitin synthase [EiChS1,2]) and of the genes encoding EiJacob1 and 3 and EiJessie1b in normal encysting cells and CS-induced differentiating cells. Expression levels are shown as fold changes at the indicated time points after the induction relative to the level at time 0 h. Representative data are shown with error bars (variation from the mean) from duplicates of two independent experiments. SPT, serine palmitoyltransferase, 1st enzyme; KDHR, 3-dehydrosphinganine reductase, 2nd enzyme; CerS, ceramide synthase, 3rd enzyme. (D to N) Changes in ceramide and major lipid species profiles in CS-induced differentiating cells at 24 h; LC-MS/MS signal intensity levels of ceramide (D), PE-Cer (E), PI-Cer (F), SM (G), PC (H), PE (I), PS (J), PI (K), LPC (L), LPE (M), and LPS and LPI (N) are shown as the fold change relative to the level at time 0 h (trophozoites). Representative data are shown from three independent experiments. Ei, *E. invadens*; PE-Cer, ceramide phosphoethanolamine; PI-Cer, ceramide phosphoinositol; SM, sphingomyelin; PC, phosphatidylcholine; PE, phosphatidylethanolamine; PS, phosphatidylserine; PI, phosphatidylinositol; LPC, lysophosphatidylcholine; LPE, lysophosphatidylethanolamine; LPS, lysophosphatidylserine; LPI, lysophosphatidylinositol.

To address this issue, the effect of CS on *de novo* Cer synthesis in CS-induced differentiating cells was investigated. Cells were metabolically labeled in the presence of different concentrations of CS using L-(U-^14^C) serine, a substrate for the first enzyme (serine palmitoyl transferase [SPT]) in *de novo* Cer synthesis. During standard encystation, accumulation of cell-associated radiolabeled Cer was observed (encystation in Fig. 3A and B) as previously shown (22). Similarly, radiolabeled Cer accumulation in CS-induced differentiating cells was also observed at levels ~2.5-fold higher than the DMSO control (Fig. 3A and B). Consistently, the transcription levels of *EiCerS5* and *6* genes, which are responsible for very-long-chain Cer-NDS synthesis, were upregulated in CS-induced differentiating cells, but that of *EiCerS2*, the other gene involved, was not (Fig. 3C). Furthermore, consistent with the cyst wall not being synthesized in CS-induced differentiating cells (Fig. 1E), the transcription levels of the genes encoding the enzymes involved in chitin metabolism, e.g., chitinases 1 to 4 (Cht1 to 4) and chitin synthase (ChS1, 2), were not upregulated. The transcription profiles of the genes encoding cyst wall proteins, such as Jacob1 and 3 and Jessie1b, in CS-induced differentiating cells were also very similar to those in DMSO-treated control cells. These results indicate that CS stage specifically and dose dependently elevates the capacity for *de novo* Cer synthesis during *Entamoeba* encystation, in which transcriptional control is involved.

We then determined Cer subclasses, which were upregulated in CS-induced differentiating *E. invadens* cells. An untargeted lipidomic analysis showed that very-long-chain Cer-NDS species, such as Cer 18:0;2O/28:2, Cer 18:0;2O/28:3, and Cer 18:0;2O/30:3, were dose dependently increased by around 5- to 30-fold at 24 h in CS-induced differentiating cells (Fig. 3D). In contrast, levels of ceramide phosphoethanolamine (PE-Cer) and ceramide phosphatidylinositol (PI-Cer), the precursor of which is Cer-NDS, in CS-treated cells were comparable to those in DMSO control cells (Fig. 3E and F), indicating that these lipids are not required for increases in membrane permeability. Meanwhile, levels of lysophosphatidylcholines (LPCs), lysophosphatidylethanolamines (LPEs), lysophosphatidylserines (LPSs), lysophosphatidylinositols (LPIs), and phosphatidylethanolamines (PEs) containing an acyl moiety with a short chain length were dose dependently increased (Fig. 3I, L, and M). In contrast, levels of phosphatidylserines (PSs) and phosphatidylinositols (PIs) were decreased (Fig. 3J and K). Importantly, CS-dependent increases in the levels of very-long-chain Cer-NDSs were abolished by 1 μM myriocin, which halts *de novo* ceramide biosynthesis in *Entamoeba* (22), while those of the lysophospholipids and phospholipids observed above were not (blue bars in Fig. 3D to F and I to N). Furthermore, all the lipid profiles observed were very similar to those seen during standard *Entamoeba* encystation (22).

**FIG 4:**
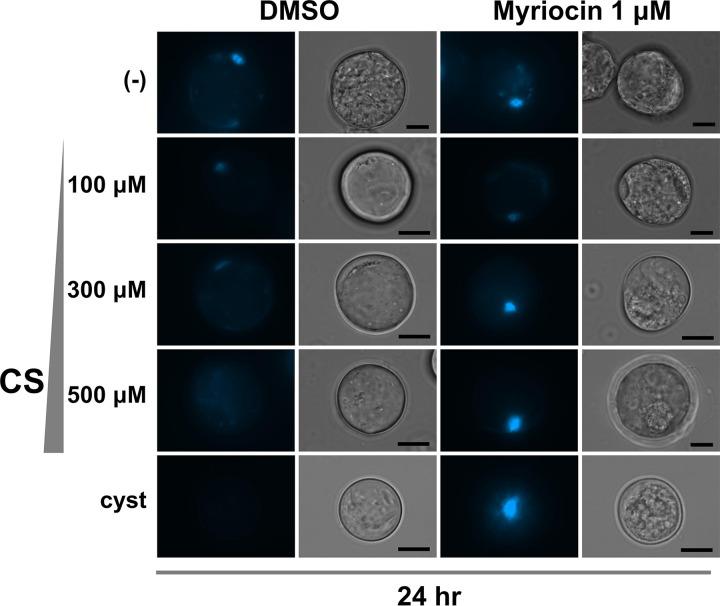
CS-mediated decrease in membrane permeability was abolished by 1 μM myriocin. Cells were differentiated in modified LG (–) with different concentrations of CS in the presence of 1 μM myriocin or 0.1% DMSO (vol/vol). Bar indicates 10 μm. Representative data are shown from three independent experiments.

To visualize the effect of 1 μM myriocin on CS-mediated membrane impermeability, differentiating cells induced by different concentrations of CS in the presence of 1 μM myriocin were stained with Hoechst 33342 at 24 h postinduction (Fig. 4). The CS-dependent membrane impermeability (DMSO in Fig. 4; see also Fig. 1C and D) was impaired by 1 μM myriocin treatment (myriocin in Fig. 4), indicating that the decrease in CS-mediated membrane permeability requires *de novo* synthesis of very-long-chain Cer-NDSs in encysting *Entamoeba* cells.

Taken together, these results indicate that CS stage specifically and dose dependently initiates and maintains the spherical shape of encysting *Entamoeba* cells. Furthermore, CS also generates cell membrane impermeability with enrichment of very-long-chain Cer-NDSs, in which transcriptional control is involved.

## DISCUSSION

**FIG 5:**
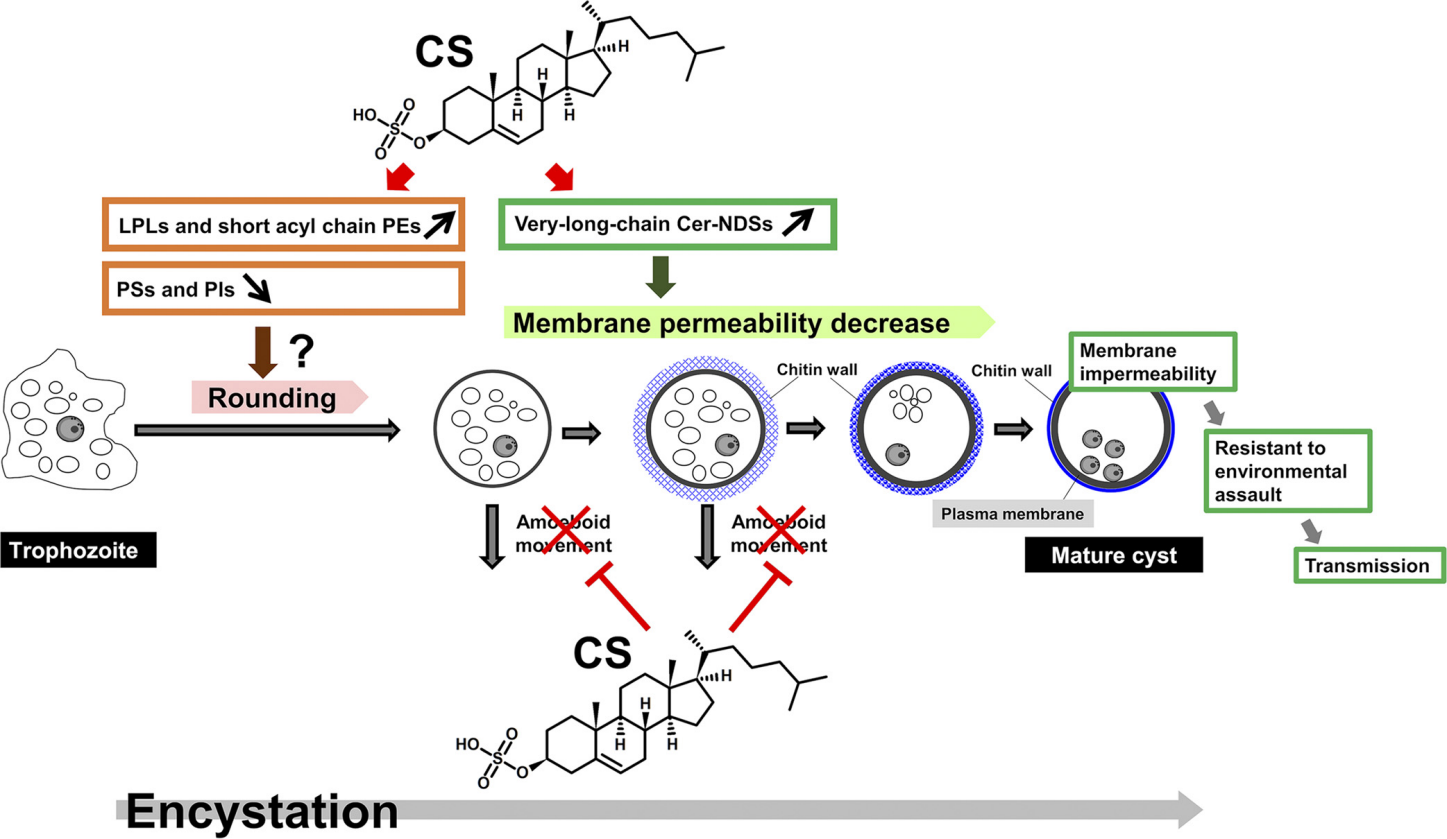
Schematic illustration of pleiotropic roles of CS in morphological and physiological changes during *Entamoeba* encystation: initiation and maintenance of the spherical shape of encysting cells and development of membrane impermeability. LPLs, lysophospholipids; PEs, phosphatidylethanolamines; PIs, phosphatidylinositols; PSs, phosphatidylserines.

Alternation of form between proliferative trophozoite and dormant cyst is a parasitic strategy that is essential for maintaining the life cycle of *Entamoeba* species. CS is crucial for the regulation of *Entamoeba* encystation, the transition stage in which trophozoites differentiate into cysts (10). However, the precise roles of CS have not been unraveled. Here, we provide distinct lines of evidence for the pleiotropic roles of CS in cell morphological and physiological alternations during *Entamoeba* encystation: initiation and maintenance of the spherical shape of encysting cells and development of membrane impermeability (see Fig. 5).

Mature *Entamoeba* cysts are round and coated with a rigid cyst wall that resists environmental assaults (see Fig. 1F). Becoming a spherical cell, a prominent phenomena during *Entamoeba* encystation, is a prerequisite for acquiring such resistance. The formation of the typical abnormal pot-like structure during cyst wall formation was previously assumed to be generated by the amoeboid movement of the encysting cell within an incomplete cyst wall (18). In this study, we show that addition of CS to encystation-inducing cultures not only initiates and maintains the spherical shape of *E. invadens* cells, but also impairs the formation of pot-like structures, resulting in increased efficiency of cyst formation. Therefore, we hypothesize that during encystation, *Entamoeba* regulates the intracellular concentration of CS to make encysting cells become rounded and nonmotile. Consequently, mature cysts are surrounded by a rigid cyst wall. The intracellular CS concentration is estimated to range from 200 (trophozoite) to 600 (cyst) μM throughout the *E. invadens* life cycle, while that of human plasma is 3.7 to 6.9 μM (15). Considering that *Entamoeba* is thought to have no capacity for *de novo* cholesterol synthesis (16), it is very likely that *Entamoeba* synthesizes the CS required for encystation via a salvage pathway using cholesterol from the host. However, the concentrations of CS and its precursor, cholesterol, in the host large intestine need to be determined.

An important and interesting question then arises: how does CS make *Entamoeba* cells rounded and decrease phagocytosis activity? We hypothesize that CS-mediated cell rounding decreases membrane fluidity, rendering its structure rigid. The potential lipid species responsible for this change in membrane fluidity, which plausibly affect membrane trafficking, were identified by our untargeted lipidomic analysis. The levels of Cer-NDSs, lysophospholipids, and PE containing an acyl moiety with a short chain length were significantly increased in a CS-treatment-specific manner, while those of PSs and PIs were decreased. Importantly, these fluctuations were similarly observed during the process of becoming rounded in normal *E. invadens* cyst formation (22). Among these lipid classes, lysophospholipids are a strong candidate for involvement in cell rounding because lysophospholipids have an inverted conical shape, which can contribute to membrane curvature (23). Although the tight packing of ceramide molecules causes a negative curvature (24), newly synthesized Cer-NDSs are unlikely to be responsible because myriocin treatment did not affect cell rounding. Of note, the elevation of lysophospholipid levels was not affected by myriocin treatment.

Development of cell membrane impermeability via *de novo* synthesis of very-long-chain Cer-NDSs was shown here to be another role of CS during *Entamoeba* encystation based on the following distinct lines of evidence: (i) elevation of the capacity for *de novo* Cer synthesis, (ii) accumulation of very-long-chain Cer-NDSs, and (iii) development of membrane impermeability that is myriocin sensitive. Notably, the specific contribution of CS to membrane impermeability was determined using a modified LG (–), a serum-free medium, with different concentrations of CS. Meanwhile, this system also showed that the accumulation of very-long-chain Cer-NDSs via *de novo* synthesis is accompanied by increased levels of *CerS5* and *6* transcription, leading us to hypothesize that CS directly or indirectly upregulates the transcription of these genes. The molecular mechanism underlying the gene regulation mediated by CS is worth further investigation. Moreover, cells induced to differentiate in this system showed decreased phagocytosis activity, a typical characteristic of normal cysts (17). This result indicates that the modified LG (–) supplemented with 100 to 500 μM CS, a cholesterol (the precursor of CS)-free medium, mimics the early phase of natural *Entamoeba* encystation. Therefore, this culture system will be useful for the study of early-phase *Entamoeba* encystation.

In a standard encystation-inducing culture, cells becoming nonmotile are likely to sequentially become sphere-shaped, have decreased membrane permeability, and form cyst wall, and the morphological and physiological changes naturally precede completion of the rigid cyst wall (17, 18, 22). The above-described system was able to differentiate cells becoming nonmotile from rigid cyst wall formation, indicating that the CS-dependent morphological and physiological changes are controlled separately from cyst wall formation. A question that remains to be answered is how CS-mediated cell morphological (becoming sphere-shaped) and physiological (decrease of phagocytosis activity and membrane permeability) changes are coordinately regulated. Meanwhile, cyst wall formation did not proceed in modified LG (–) supplemented with an optimum concentration of CS but was completed by addition of ABS to the medium. Therefore, serum components other than CS, which need to be determined, may exert an important role in regulating cyst wall formation.

In conclusion, this study provides evidence that CS is a multifunctional molecule with pleiotropic roles during *Entamoeba* encystation (Fig. 5). This study also addresses dormancy as a parasitic strategy and control of membrane structure and vesicular trafficking in *Entamoeba*, all of which warrant further investigation to gain a greater understanding of *Entamoeba* physiology and general cell and lipid biology.

## MATERIALS AND METHODS

### Medium.

Three types of medium were used, each for a different purpose.

1. BI-S-33 medium, the composition of which is described in reference 25, for the routine culture of *E*. *invadens* (IP-1).
2. 44% LG medium supplemented with 3% ABS as a standard encystation-inducing medium for *E*. *invadens* (IP-1).
3. Modified LG medium (modified LG [–]) supplemented with different concentrations of CS or 500 μM PC for investigating the effect of CS or PC on *E*. *invadens* (IP-1) cells.

LG medium composition was as described in reference 14 and diluted to 44% with sterilized deionized water. Modified LG (–) was 15.8 mM glucose in 44% LG medium. CS and PC were purchased from Sigma-Aldrich (catalog no. C9523 and P3556, respectively; St. Louis, MO, USA), dissolved in dimethyl sulfoxide (DMSO) and ethanol, respectively, at 50 mM as a stock solution, and kept at −30°C.

### Parasite culture and encystation induction.

*E*. *invadens* (IP-1) was routinely maintained in a glass tube filled with 6 mL BI-S-33 medium (proliferation medium; see the previous section) (22). To induce encystation, stationary-phase trophozoites were harvested and then transferred to a standard encystation-inducing culture medium (see the previous section) at 6 × 10^5^ cells/mL. Stationary-phase trophozoites were prepared by cultivation in proliferation medium for 5 days with an inoculum size of 1 × 10^4^ cells/mL using cells from the routine culture, reaching a cell density of ~2.4 × 10^5^ cells/mL.

### Quantification of CS in *E*. *invadens* trophozoites and cysts.

Stationary-phase trophozoites, prepared as described above, were adjusted to 1 × 10^6^ cells and used as trophozoite samples. Cyst samples from standard encystation-inducing cultures (see the previous section) at 72 h postinduction were adjusted to 1 × 10^6^ cells. Lipids from each sample were extracted as previously described (22). The obtained lipids were suspended in 1 mL methanol (MeOH)/CHCl_3_/H_2_O solution (2:1:0.2 [vol/vol/vol]) and kept at 4°C until use. As a standard stock solution, CS (see “Medium”) was dissolved in MeOH/CHCl_3_/H_2_O at 2 mM and kept at −30°C.

CS concentration was determined by measuring the corresponding peak area in the chromatogram generated using a tandem LC-MS/MS system; the standards were prepared by diluting a 2 mM stock with MeOH/CHCl_3_/H_2_O. The high-performance liquid chromatography system was equipped with a Shim-pack XR-ODSIII column (150 × 2.0 mm; 2.2 μm) coupled with a Shimadzu LCMS-8030 triple quadrupole (Shimazu Co. Ltd., Kyoto, Japan). The liquid chromatography apparatus consisted of a DGU-14 AM degasser, an LC-30AD pump, a CTO-10Avp column oven, and a SIL-30AC auto-sampler (Shimazu). Mobile phase A was acetonitrile/methanol/water (1:1:3 [vol/vol/vol]) containing 5 mM ammonium acetate and 10 nM EDTA. Mobile phase B was 100% isopropanol containing 5 mM ammonium acetate and 10 nM EDTA. The gradient was 0 min, 0% B; 1 min, 0% B; 5 min, 40% B; 7.5 min, 64% B; 12 min, 64% B; 12.5 min, 82.5% B; 19 min, 85% B; 25 min, 95% B; 25.1 min, 0% B; and 30 min, 0% B. The flow rate was 0.2 mL/min, the temperatures of the column and autosampler were 47 and 4°C, respectively, and the injection volume was 5 μL. The electrospray ionization (ESI) source was operated with the following conditions: nebulizing gas flow, 3.0 L/min; drying gas flow, 15 L/min; desolvation line (DL) temperature, 250°C; heat block temperature, 400°C. Analysis was performed in negative ion mode and with multireaction monitoring. Optimal precursor ions (*m/z*), collision energy (CE), the first quadrupole (Q1) and third quadrupole Q3 prebias rod voltages (V), and product ions (*m/z*) were optimized using the LabSolutions (v5.99 SP2) Optimization Wizard function. To fulfill all optimization parameters, 2 μL of 2 μM CS standard solution was injected. The observed transition in multireaction monitoring was from 465.3 to 97.0 *m/z*, corresponding to the [M-H]^−^ precursor ion and product ion, respectively. Q1 voltage, Q3 voltage, signal collection time (dwell time), and CE were −13.0 V, −12.0 V, and 100 msec, and −41.0 V, respectively.

### Cell differentiation induced by CS.

Stationary-phase trophozoites were transferred to modified LG (–) (see “Medium”) containing different concentrations of CS (0, 100, 300, and 500 μM) at 6 × 10^5^ cells/mL and then cultivated for the periods indicated in Fig. 1, 3, and 4 in either the absence or presence of 1 μM myriocin. PC at 500 μM was used as a negative control.

### Fluorescence microscopy for CS-induced differentiating cells.

To assess phagocytosis activity, the uptake of latex beads by differentiating *Entamoeba* cells, induced by 500 μM CS treatment for 24 h, was assessed as described in reference 26. As reference controls, trophozoites and cysts from routine and standard encystation cultures, respectively (see “Medium”), were used. For latex bead uptake, 10 μL of a fluorescent red carboxylate-modified polystyrene 2.0-μm latex bead suspension (L3030; Sigma-Aldrich) was mixed with 100 μL phosphate-buffered saline (PBS), and the beads were pelleted by centrifugation at 2,380 × *g* for 5 min at room temperature. The pellet was resuspended in 10 μL PBS and added to 240 μL of each culture, and culturing was continued. Then, 3 h later, cells were pelleted from each culture by centrifugation at 2,000 × *g* for 15 sec at room temperature and washed with 500 μL PBS three times. The obtained cell pellet was then resuspended in 30 μL PBS and analyzed under a fluorescence microscope (Zeiss Axio Imager 2; Carl Zeiss, Germany) equipped with an AxioCam 305 mono camera (Carl Zeiss). The obtained images were processed using ZEN software (Carl Zeiss).

For actin staining, cells from two wells of a 96-well culture plate were collected into a single 1.5-mL tube containing 300 μL PBS, pelleted by centrifugation at 770 × *g* for 1 min at room temperature, and fixed with 4.0% paraformaldehyde in PBS for 15 min at room temperature. The fixed cells were permeabilized with 0.2% Triton X-100 in PBS for 15 min and then blocked with 2% BSA in PBS for 30 min at room temperature. Cells were then repelleted and suspended in 300 μL PBS containing 1 μg/mL Hoechst 33342 (Sigma-Aldrich) together with 6.6 μM Alexa Fluor 488 phalloidin. Prepared cells were observed and images were processed as described above.

For analysis of cell roundness, stationary-phase trophozoites were cultured in the presence of 1 μM CellTracker orange CMRA {9’-(4 [and 5]-chloromethyl-2-carboxyphenyl)-7’-chloro-6’-oxo-1,2,2,4-tetramethyl-1,2-dihydropyrido[2’,3’-6]xanthene} dye from Invitrogen (catalog no. C34551; Waltham, MA, USA) for 1 h and then transferred to a different culture medium. Then, 24 h later, cells were pelleted by centrifugation and suspended in PBS. Cell suspensions from different cultures were observed under a fluorescence microscope as described above, and the obtained images were processed using ImageJ (https://imagej.nih.gov/ij/) to calculate the value of cell roundness. At least 20 cells from each sample were analyzed.

To analyze for the cyst formation and the development of membrane impermeability, cells from two wells of a 96-well culture plate were collected for the times and conditions indicated in Fig. 1 and 4 into a single 1.5-mL tube containing 300 μL phosphate-buffered saline (PBS). Cells were pelleted by centrifugation at 770 × *g* for 1 min at room temperature. Cell pellets were then suspended in 300 μL PBS containing 1 μg/mL Hoechst 33342 (Sigma-Aldrich) alone or together with 10 μg/mL FITC-conjugated wheat germ agglutinin (WGA; Sigma-Aldrich). Prepared cells were observed and images processed as described above.

### Counting the different types of cyst wall structures and flow cytometry.

Encystation-inducing cells prepared as described in (“Parasite Culture and Encystation Induction”) were cultivated in a standard encystation-inducing medium (medium no. 2 in “Medium”) for 16 or 24 h in the presence of different concentrations of CS and D-B-09 (indicated in Fig. 2). For counting the three types of cyst wall structure, i.e., pot-like, irregularly shaped, and round, cell pellets prepared from two wells of a 96-well culture plate as described above were suspended in 300 μL 5-fold-diluted calcofluor white stain (Sigma-Aldrich) solution (in PBS). Following cell treatment, counts were performed as previously described (18). Flow cytometry using two dyes, Evans blue and calcofluor, was performed as described previously (27).

### Metabolic labeling of *E*. *invadens* and lipid analysis.

*E. invadens* trophozoites suspended at 6 × 10^5^ cells/mL either in a standard encystation-inducing medium or modified LG (–) (see “Medium”) supplemented with 0, 300, or 500 μM CS were seeded in 96-well culture plates (240 μL per well). After adding [^14^C(U)] l-serine (173.6 mCi/mmol) (Moravek, Brea, CA, USA) to each well (final radioactivity, 3 μCi/mL), the plates were sealed and incubated at 26°C for 24 h. Cell cultures from six wells were collected into a glass tube containing 2 mL PBS and centrifuged at 770 × *g* for 1 min at room temperature. Following lipid extraction and thin-layer chromatography, analysis of each lipid was performed as previously described (22).

### Real-time qRT-PCR.

Real-time reverse transcription-quantitative PCR (qRT-PCR) was performed as previously described (22) using appropriate primer sets listed in Table S1 in the supplemental material and reference 22.

10.1128/msphere.00299-22.1 TABLE S1 Primers used in this study. Download Table S1, TIF file, 0.7 MB. Copyright © 2022 Mi-ichi et al. 2022 Mi-ichi et al. https://creativecommons.org/licenses/by/4.0/ This content is distributed under the terms of the Creative Commons Attribution 4.0 International license.

### LC-MS/MS-based untargeted lipidomics.

The samples for untargeted lipidomics were prepared as described in reference 22, and the LC-MS/MS analysis and data processing methods were conducted as described previously (28).

### Data availability.

All raw mass spectrometry data are freely available on the RIKEN DROP Met website (http://prime.psc.riken.jp/menta.cgi/prime/drop_index), under index number DM0045.
