# Supplementary figures and images for: Pleiotropic Roles of Cholesteryl Sulfate during Entamoeba Encystation: Involvement in Cell Rounding and Development of Membrane Impermeability
Source: mSphere. 2022 Aug 9;7(4):e00299-22. doi: 10.1128/msphere.00299-22 (PMC9429911; doi:10.1128/msphere.00299-22)

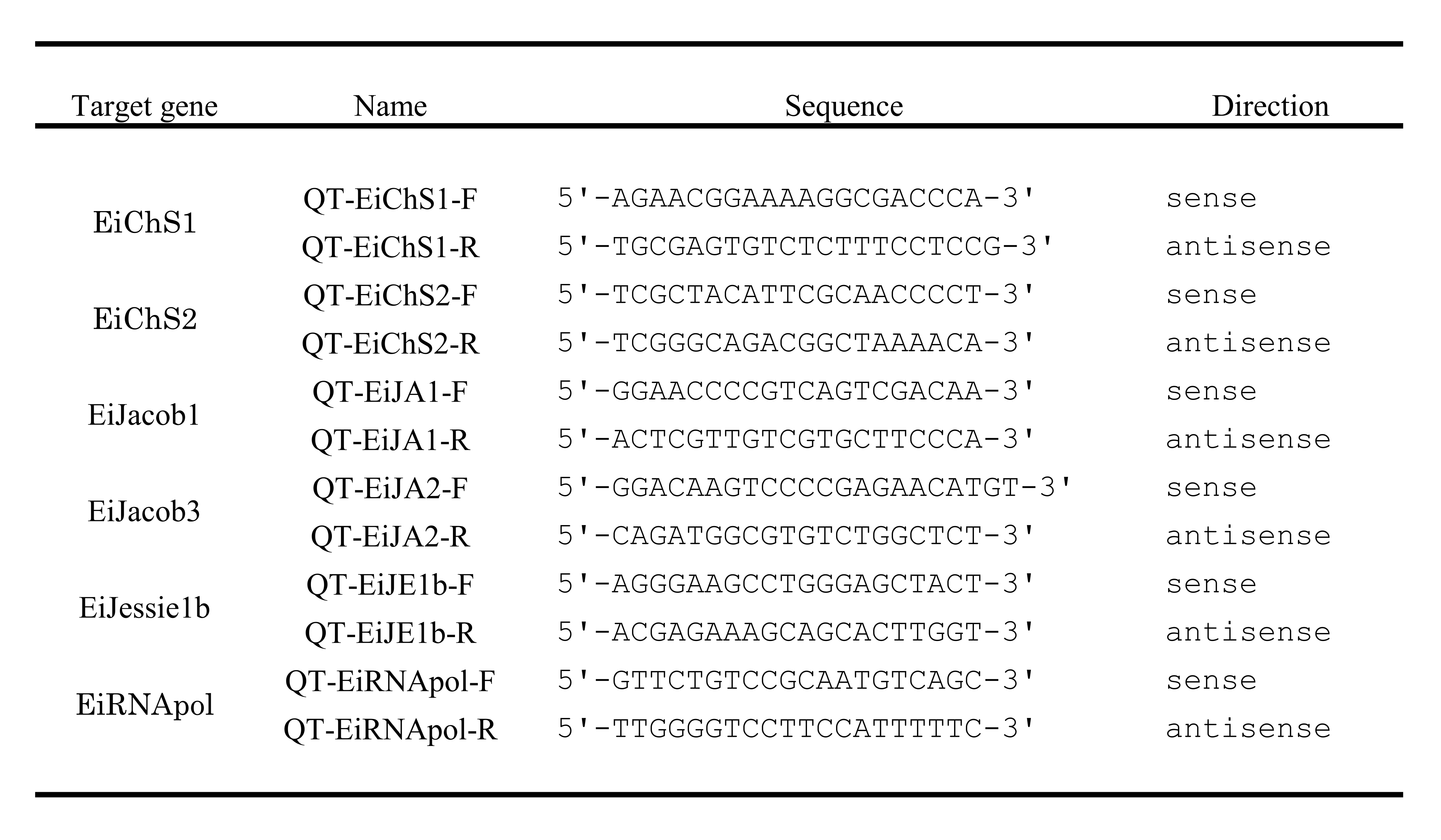

Supplement: TABLE S1 [file msphere.00299-22-s0001.tif]
